# Estimating Respiratory Rate in Post-Anesthesia Care Unit Patients Using Infrared Thermography: An Observational Study

**DOI:** 10.3390/s18051618

**Published:** 2018-05-18

**Authors:** Nadine Hochhausen, Carina Barbosa Pereira, Steffen Leonhardt, Rolf Rossaint, Michael Czaplik

**Affiliations:** 1 Department of Anesthesiology, Medical Faculty, RWTH Aachen University, 52074 Aachen, Germany; rrossaint@ukaachen.de (R.R.); mczaplik@ukaachen.de (M.C.); 2 Philips Chair for Medical Information Technology, Helmholtz Institute for Biomedical Engineering, RWTH Aachen University, 52074 Aachen, Germany; pereira@hia.rwth-aachen.de (C.B.P.); leonhardt@hia.rwth-aachen.de (S.L.)

**Keywords:** infrared thermography, breathing rate detection, long-wave infrared

## Abstract

The post-anesthesia care unit (PACU) is the central hub for recovery after surgery, especially when the surgery is performed under general anesthesia. Aside from clinical aspects, respiratory impairment is one of the major causes of morbidity and affected recovery in the PACU and should therefore be monitored. In previous studies, infrared thermography was applied to assess the breathing rate (BR) of healthy volunteers. Here, the transferability of published methods for postoperative patients in the PACU was examined. Video recordings of 28 patients were acquired using a long-wave infrared camera, and analyzed offline. For validation purposes, BRs derived from body surface electrocardiography were measured simultaneously. In general, a close agreement between the two techniques (r = 0.607, *p* = 0.002 upon arrival, and r = 0.849, *p* < 0.001 upon discharge from the PACU) was obtained. In conclusion, the algorithm was demonstrated to be feasible and reliable under these challenging conditions.

## 1. Introduction

The post-anesthesia care unit (PACU) is the central hub for recovery after surgery, especially when the surgery is performed under general anesthesia [1]. During stays in the PACU, patients have to be monitored (clinically, and by assessing vital parameters) and stabilized in terms of potential volume deficits, pain, and body temperature, if necessary, before being transferred to a normal unit [2]. Respiratory complications are a major cause of morbidity and impaired recovery in the PACU, as respiratory function is already affected by general anesthesia. Thus, impaired oxygenation and a reduction of up to 50% in functional residual capacity when compared with pre-anesthesia values were reported. In addition, various critical respiratory events (CREs) can occur during stays in the PACU, including hypoxemia, where one’s oxygen saturation (SpO_2_) lies below 90%; hypoventilation, where one’s breathing rate (BR) rests below 8 breaths per minute (bpm), or one’s partial pressure of carbon dioxide in arterial blood (pCO_2_) rises above 50 mmHg; and upper airway obstruction. The factors contributing to these respiratory complications can be patient-, surgical-, or anesthesia-related. Therefore, early detection of these CREs is very important for a prompt and appropriate medical intervention, such as a physical treatment (e.g., insertion of an oral or nasal supraglottic airway device, oxygen insufflation, or even tracheal reintubation) or pharmacological therapy (e.g., opioid antagonism or muscle relaxant reversal) [3,4]. In addition, the monitoring of breathing function is essential because altered breathing patterns such as tachypnea (high BR), bradypnea (low BR), apnea (suspension of breathing), or irregular breathing (e.g., Cheyne–Stokes respiration or Kussmaul breathing) may indicate impaired recovery [4,5,6,7]. For these reasons, BR and other vital parameters, such as heart rate and temperature, should be assessed continuously in PACUs.

Although BR is a solid indicator, and one of the earliest indicators of physiological distress, studies have reported that it is also commonly neglected due to the shortcomings of current clinical monitoring techniques [8]. The current methods used to monitor vital signs require the attachment of sensors to the patient’s body, which frequently leads to discomfort and stress [9]. Therefore, the demand for contactless monitoring techniques in clinical settings is increasing, not only for vital parameter detection, but also for diagnostic tools [10,11,12,13,14,15]. Several research groups have demonstrated that infrared thermography (IRT) may be a promising technology for the monitoring of BR [16], heart rate [17], temperature [18], and perfusion [19]. It combines various advantages, such as passive, contactless, and radiation-free measurement procedures, as well as independence from a light source [19].

In 2015, a robust and effective algorithm for BR detection in thermal videos was introduced by Pereira et al. It ensured automatic detection and tracking of the nose, which was the region of interest (ROI). Furthermore, successful extraction and processing of breathing waveforms was demonstrated in an experimental study involving healthy volunteers [20]. We strongly believe that PACU patients may benefit from contactless BR estimation. Therefore, in this paper, the performance of the approach developed by Pereira et al. was evaluated in terms of reliability and feasibility on postoperative patients in the PACU. To the best of our knowledge, this is the first study in which IRT was used to monitor BR in a real clinical scenario involving adult patients.

## 2. Materials and Methods

### 2.1. Study Design

All subjects gave their informed consent for inclusion before participating in the study. The study was conducted in accordance with the Declaration of Helsinki, and the protocol was approved by the Ethics Committee of the RWTH Aachen Faculty of Medicine, Germany (EK 041/14). The observational study was performed in the PACU from January 2016 to April 2016 at the University Hospital Aachen. 

Twenty-eight patients receiving general anesthesia for non-emergency surgeries were enrolled in this observational study. Inclusion criteria were age over 18 years, the ability to give written consent, and a postoperative stay planned in the PACU. Exclusion criteria resulted from the inclusion criteria: underage patients, the inability to give written consent, and a postoperative stay planned in the intensive care unit or intermediate care unit.

### 2.2. Monitoring and Data Assessment

After admission to the PACU, all patients were connected to a Philips IntelliVue MP30 monitor (Philips Electronics N.V., Amsterdam, The Netherlands) for continuous assessment of vital data (electrocardiography (ECG), noninvasive blood pressure, BR, and oxygen saturation). 

According to clinical standards, vital signs were collected and documented every 15 min. Additionally, a 2-minute IRT video (after calibration) was acquired in 15-minute phases, focusing on the patient’s face. Vital signs were recorded simultaneously. However, in this study, only two thermal videos were analyzed per patient. These corresponded to the first (upon admission to the PACU) and last measurements (upon discharge from the PACU). 

IRT sequences were recorded with an uncooled, long-wave infrared camera, VarioCAM^®^ HD head 820 S/30 mm (Infratec GmbH, Dresden, Germany), using the software IRBIS 3 plus. Its spatial resolution covers 1024 × 768 pixels, the spectral range is between 7.5 μm and 14 μm, and the thermal sensitivity is 50 mK at 30 °C. In addition, sequences were acquired at a rate of 30 frames per second.

**Figure 1:**
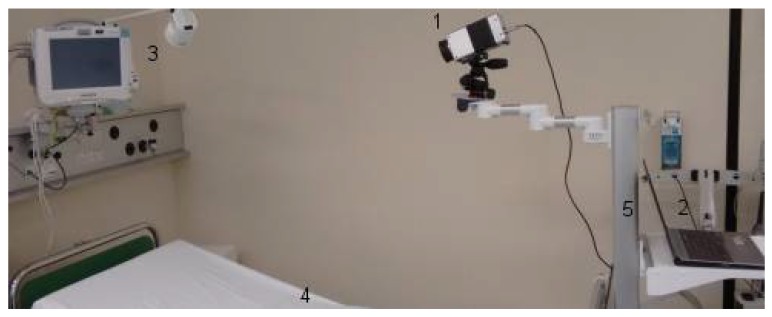
The experimental setup in the post-anesthesia care unit (PACU). (1) Long-wave infrared camera, VarioCAM^®^ HD head 820 S/30 mm (Infratec GmbH, Dresden, Germany). (2) Laptop. (3) Philips IntelliVue MP30 monitor (Philips Electronics N.V., Amsterdam, the Netherlands). (4) Patient bed. (5) Rolling rack.

The experimental setup, assuring quality of data acquisition, was placed in the PACU, which was air-conditioned. The infrared camera was mounted on a tripod head, which was fixed on a rolling rack. The rolling rack was positioned at the foot of the bed. All patients laid in the bed with an elevated upper body, wearing patient hospital clothing and no jewelry. All patients were sober after their surgical procedures (Figure 1).

### 2.3. IRT Image Analysis

**Figure 2:**
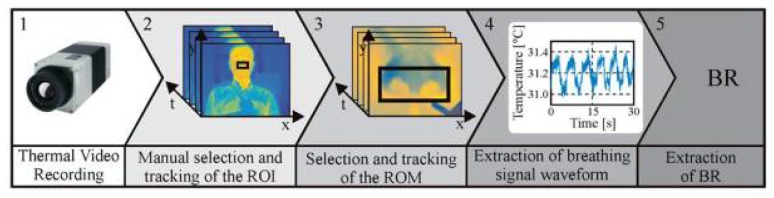
Representation of the five major steps used to extract breathing rate (BR) from thermal videos. (1) Video acquisition. (2) Manual selection and tracking of the nose, which is the region of interest (ROI). (3) Manual selection and tracking of the region of measurement (ROM), which encloses the nostrils. (4) Extraction of the breathing waveform. (5) Breathing rate estimation.

The approach used to assess BR relies on the temperature variation around the nose during the respiratory cycle. During inspiration, cold air from the environment is inhaled, and during expiration, warm air from the lungs is exhaled. As demonstrated in several pilot studies [16,20,21,22], thermal imaging is capable of accurately detecting this temperature modulation. Figure 2 shows the main steps used to assess BR from thermal videos. These are explained in the following section in more detail. As described by Pereira et al. [20], the algorithm was implemented in MATLAB (MATLAB 2014a, The MathWorks Inc., Natick, MA, USA). The collected data were analyzed offline, using a standardized protocol.

#### 2.3.1. Region Selection and Tracking

The first step of this approach consisted of manually selecting the ROI (i.e., the nose of the patient). Afterward, a rough tracking of the nose was carried out using the algorithm proposed by Mei et al. [23]. It is a particle filter-based object tracker that integrates sparse representation. The tracking algorithm considers two models: a state transition model, $p\left(x_{t}{|x}_{t-1}\right)$, and an observation model, $p(y_{t}|x_{t})$. The former, $p(x_{t}|x_{t-1})$, uses conditional density to compute the temporal correlation of a state transition between two consecutive frames. Here, $x_{t}$ is calculated using the affine transformation parameters $\left(\alpha_{1},\alpha_{2},\alpha_{3},\alpha_{4},t_{1},t_{2}\right)$, and velocities $\left(v_{1},v_{2}\right)$, where $\left(\alpha_{1},\alpha_{2},\alpha_{3},\alpha_{4}\right)$ are the deformation parameters, and $\left(t_{1},t_{2}\right)$ stand for the translation parameters, which describe displacement along the two axes, x and y. Finally, $\left(v_{1},v_{2}\right)$ correspond to the velocities of both translation parameters. The latter, $p(y_{t}|x_{t})$, compares the similarity between the target model and target candidate at each time, *t*. The maximum observation likelihood is given by Equation (1):

(1) $$p\left(y_{t}{|x}_{t}\right)=\underset{j=1,\ldots ,d}{\prod }N\left({\tilde{y}}_{t}\left(j\right);0,\sigma^{2}\right).$$

Here, $y_{t}$ is the observation, N(·) stands for the Gaussian distribution, and $\sigma^{2}$ represents its variance. Additionally, j denotes the jth pixel, d is the number of pixels in the appearance model, and ${\tilde{y}}_{t}$ stands for the approximation residual of the observation, $y_{t}$ by the target model.

To improve the signal-to-noise ratio, a second ROI, labeled the region of measurement (ROM), was defined and tracked.

#### 2.3.2. Extraction of the Respiratory Waveform and Signal Processing

After the tracking procedure, the mean temperature value, $\bar{s}\left(t\right)$, of the ROM was calculated for each frame of the video according to Equation (2):

(2) $$\bar{s}\left(t\right)=\frac{1}{m\cdot n}\;\overset{m-1}{\underset{i=0}{\sum }}\overset{n-1}{\underset{j=0}{\sum }}s\left(i,j,t\right).$$

In this equation, m and n stand for the width and length of the ROM, and s(i,j,t) corresponds to the temperature at pixel (i,j) of frame t. The variable $\bar{s}\left(t\right)$ represents the temperature waveform around the nostrils. The next step was its preprocessing, by applying a second-order Butterworth bandpass filter.

To estimate the instantaneous respiratory frequencies, the approach presented by Brüser et al. was used [24]. Firstly, a short adaptive analysis window, $w_{i}\left[v\right]$, centered at $n_{i}$, was slid across the respiratory signal, s[n]. Secondly, for each window position, the local breath-to-breath interval, $T_{i}$, was computed using three estimators: $E_{AC}\left[m\right]$, $E_{AMDF}\left[m\right]$, and $E_{MAP}\left[m\right]$. The adaptive window autocorrelation estimator, $E_{AC}\left[m\right]$, calculates, for all interval lengths, the correlation between m samples to the right, w[v], and to the left, w[v−m], of the window center, w[0], and was defined as shown in Equation (3):

(3) $$E_{AC}\left[m\right]=\frac{1}{m}\;\overset{m}{\underset{v=0}{\sum }}w\left[v\right]\cdot w\left[v-m\right].$$

The adaptive window average magnitude difference function, $E_{AMDF}\left[m\right]$, computes, in turn, the absolute difference between samples for all interval lengths as defined by Equation (4):

(4) $$E_{AMDF}\left[m\right]={\left(\frac{1}{m}\;\overset{m}{\underset{v=0}{\sum }}\left|w\left[v\right]\cdot w\left[v-m\right]\right|\right)}^{-1}.$$

The third and last estimator, the adaptive maximum amplitude pairs, $E_{MAP}\left[m\right]$, is an indirect peak detector because it calculates the maximum amplitude of any two samples for each lag, [m], as shown in Equation (5):

(5) $$E_{MAP}\left[m\right]=\underset{v\in \left\{0,\ldots ,m\right\}}{\max}\left(w\left[v\right]\cdot w\left[v-m\right]\right).$$

After computing the three estimators, Bayesian fusion was applied. Given $E_{AC}\left[m\right]$, $E_{AMDF}\left[m\right]$, and $E_{MAP}\left[m\right]$, the conditional probability of m being the correct breath-to-breath interval is governed by Equation (6):

(6) $$p\left(m|E_{AC},E_{AMDF},E_{MAP}\right)\propto p\left(m|E_{AC}\right)p\left(m|E_{AMDF}\right)p\left(m|E_{MAP}\right).$$

Note that the estimators $E_{AC}\left[m\right]$, $E_{AMDF}\left[m\right]$, and $E_{MAP}\left[m\right]$ can be regarded as probability density functions: $p\left(m|E_{AC}\right)$, $p\left(m|E_{AMDF}\right)$, and $p\left(m|E_{MAP}\right).$

### 2.4. Statistical Analysis

No power analysis was performed due to the observational nature of this study. 

All data were analyzed with SPSS Statistics 23 for Windows (SPSS Inc., IBM Business Analytics Software, Armonk, NY, USA). The statistical significance level was set at *p* < 0.05. The Kolmogorov–Smirnov test confirmed the non-normal distribution of the data. Therefore, Spearman’s rho correlation coefficients were presented for the time points “arrival at the PACU” and “discharge from the PACU” separately, in order to consider intra-individual dependencies within one dataset. 

**Figure 3:**
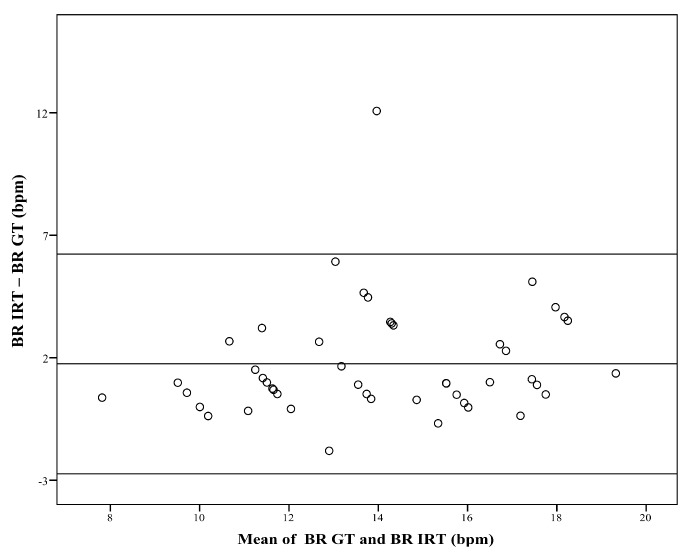
Difference against mean for breathing rate (BR). The figure compares the breathing rate (BR) obtained with infrared thermography (BR IRT) with the corresponding BR measured with the ground truth (BR GT). The plot shows a bias of 1.75 bpm, and limits of agreement range from −2.74 bpm to 6.23 bpm.

The Bland–Altman plot (Figure 3) compares the BR obtained with IRT, and the ground truth (GT, which is the BR derived from body surface ECG). For subgroup analysis, patients were assigned to category A (BR < 12 bpm), category B (BR: 12–15 bpm), or category C (BR > 15 bpm). Since the number of resulting datasets was relatively low, correlation analysis was performed over both time points.

## 3. Results

In total, 28 patients were included in the study. Thus, in this work, 56 datasets (two datasets per patient) were analyzed. No patient declined measurements after participation in the study. Forty-seven datasets were included in the data analysis. Nine datasets were unable to be analyzed and were therefore excluded, either because the ROI (nose) was not visible, or because of the patient’s constant movement. The study population had an age of 70 years (interquartile range (IQR) 51–77), and consisted of 24 female and 4 male patients.

The Bland–Altman plot (Figure 3) shows the comparison between the two measurement techniques. It compares the BR obtained with IRT (BR IRT) with the BR of the GT (BR GT). The plot shows a bias of 1.75 bpm, and limits of agreement ranged from −2.74 bpm to 6.23 bpm. 

Table 1 shows the performance of the algorithm in terms of correlation coefficients and *p*-values, for the three BR categories (A to C). On average, Spearman’s rho correlation coefficient (r) between the BR estimated using IRT, and using the GT was r = 0.607 (*p* = 0.002) upon arrival and r = 0.849 (*p* < 0.001) upon discharge from the PACU. The highest correlation was obtained for category A (BR < 12 bpm, r = 0.845, and *p* = 0.034), and the lowest performance was obtained for higher BRs (BR > 15 bpm, category C, r = 0.458, and *p* = 0.024). 

**Figure 4:**
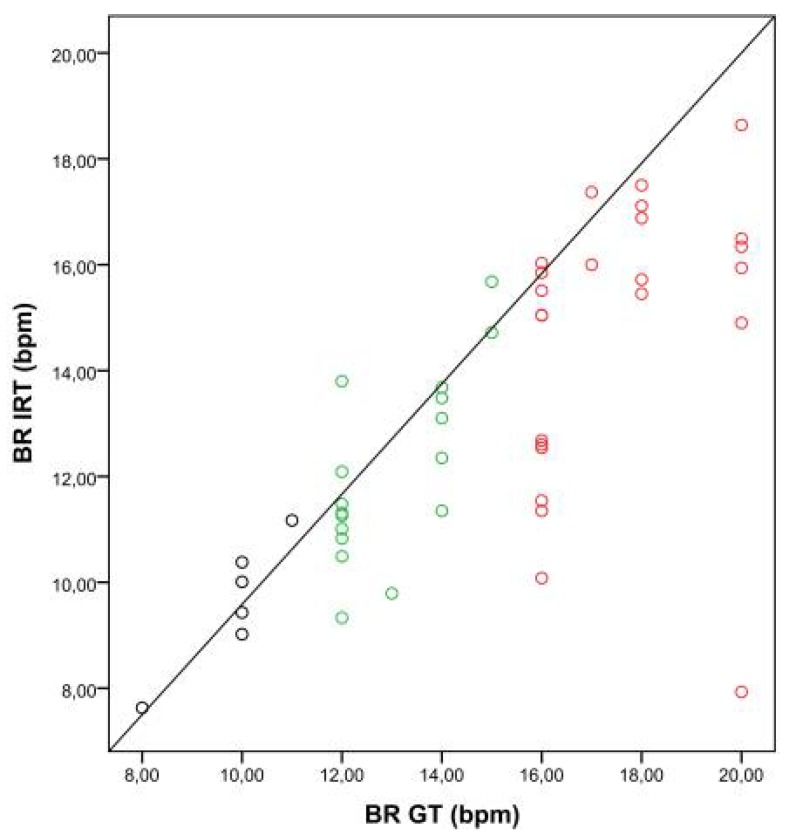
Correlation between breathing rates (BRs) obtained with the ground truth (BR GT) and IRT (BR IRT). The scatter plot shows the relationship between BRs obtained with the GT and IRT for three BR categories (A: BR < 12 bpm, black dots; B: BR 12–15 bpm, green dots; C: BR > 15 bpm, red dots).

Figure 4 shows the correlation between BRs obtained with IRT and the GT, depending on the breathing categories, A to C.

**Figure 5:**
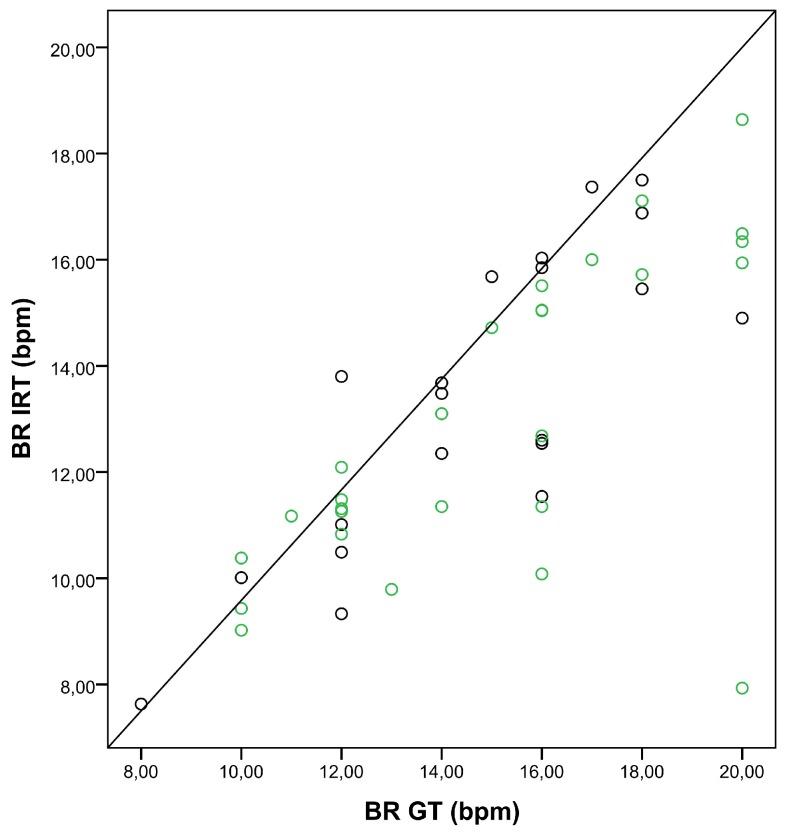
Correlation between breathing rates (BRs) obtained with the ground truth (BR GT) and IRT (BR IRT). The scatter plot shows the relationship between BRs obtained with the GT and IRT for insufflation with nasal cannula (green dots), and no insufflation (black dots).

Table 2 compares the correlation between IRT and the GT for both cases: oxygen insufflation and no insufflation. The algorithm’s performance was only slightly lower when the patient needed insufflation with a nasal cannula. Additionally, the correlation between BRs obtained with the GT and with IRT is presented in the scatter plot (Figure 5).

Assessing each patient individually, it was clear that the algorithm performed better for regular, deep breathing patterns than for shallow breathing (Table 3). 

## 4. Discussion

In this paper, the reliability and feasibility of a new algorithm for BR estimation from thermal videos of postoperative patients in the PACU was examined. The key result was that this IRT-based approach, which was previously validated in a study involving healthy volunteers, was capable of accurately assessing BR in a realistic clinical scenario, typically found in the PACU. 

In our study, thermal videos from postoperative patients staying in the PACU were acquired. The estimated BR was compared with the BR derived from body surface ECG (ground truth). A high degree of agreement between both measurement techniques was observed, even though the nostril region (ROI) represented a relatively small portion of the image (smaller than 1%). In category A (BR < 12 bpm) and category B (BR 12–15 bpm) in particular, the strength of the relationship was high. A lower correlation coefficient was observed for higher breathing frequencies (category C, BR > 15 bpm), surely influenced by outliers (Figure 4). One possible explanation may be the shallow breathing accompanying tachypnea. 

In some cases, the reference method may have failed to detect episodes of shallow breathing and bradypnea, contributing to higher errors. Because the assessment of BR was only conducted at one defined time point, and several measures were obtained from IRT sequence analysis, correlation was impaired for irregular breathing (Table 3). Broens et al. claimed that ECG-derived BR monitoring is error-prone and might lead to underdetection of respiratory events [6]. In addition, it is very prone to motion artefacts, poor electrode placement, and physiologic events that induce thoracic movements unrelated to respiration (e.g., coughing) [25]. Fortunately, no disruptive influence factors were present in the analyzed IRT sequences of this study. In total, IRT-based BR detection may be superior to ECG-derived BR monitoring in particular situations. Extreme perspiration also complicates ECG electrode attachment. Additionally, postoperative patients with reduced awareness of their situation, confused patients, or patients with senile dementia tend to remove sensors. In all these situations, remote monitoring techniques may be advantageous.

At this time, we know that the algorithm used, based on IRT, worked accurately in healthy volunteers in a laboratory setting, even under challenging conditions. Here, the healthy participants were asked to move, as well as to simulate various types of breathing disorders (e.g., Kussmaul breathing, Cheyne–Stokes respiration, tachypnea, etc.). The results of the study demonstrated that this approach was able to compensate for the patient’s movement, and to accurately estimate BR. A good agreement between IRT and piezoplethysmography (reference measurement) was reported. Additionally, the approach seemed to be robust against motion as well as various breathing patterns [20]. Overall, localization of selected features, analysis of thermal images, and image segmentation of the face are always challenging [26,27,28]. However, other approaches in the literature show promising results using IRT for BR estimation. Specifically, Lewis et al. published an IRT-based approach for the estimation of BR and relative tidal volume in participants. In this case, a variety of breathing protocols were performed and then analyzed. Similar close correlations between thermal and mechanical BRs were obtained [16]. Additionally, Fei et al. introduced a thermal imaging approach using wavelet analysis for filtering out breathing information. Here, a high correlation between the IRT approach and the reference method was also demonstrated [21]. In 2010, Al-Khalidi proposed an algorithm that also used skin temperature changes around the nose during a respiration cycle for respiration monitoring. In contrast to our approach, it computed the position of the nose (region of interest) for every single frame [29]. This method is probably computationally costly, but is less sensitive to sudden and unpredictable strong movements of the object. In 2017, Alkali presented an improved version of this approach [30]. 

In this paper, we used a more sophisticated tracking algorithm which employs a motion model describing how the target may change for various possible motions of the object. Here, the tracking always relies on prior information to estimate the new position of the object. Furthermore, Chauvin et al. demonstrated that real-time measurements during exercising are possible using a thermal camera [31]. In 2018, a case report was published showing an acceptable performance of thermal cameras for BR estimation during nighttime measurements [32]. Another study demonstrated accurate robustness, despite different amounts of thermal changes and motion [33].

Despite the high correlation between the IRT-based approach and the GT, all introduced studies worked with voluntary participants under laboratory conditions. Even if participants were instructed to perform various movements and breathing patterns during data acquisition, an experimental setting does not reflect a clinical scenario. In our study, we analyzed, for the very first time, the capability of IRT to estimate the BR of adult patients in a postoperative clinical setting. Here, the patients arrived for treatment in the PACU after a variety of surgical procedures. Postoperative pain and state of consciousness varied. Additionally, some patients needed an oxygen supply via a nasal cannula. The data recording was performed without instructions or restrictions for the patients. Nevertheless, close correlations between the IRT-based approach and the GT were obtained, despite the challenging conditions that characterize the typical clinical environment. 

As referred to in Section 2.3.1, the ROI (nose) was manually selected. Thus, our next goal is to improve the current approach to work automatically. Some pathologies, such as Raynaud’s phenomenon, must be considered since they can negatively affect the segmentation of the ROI (patients suffering from this syndrome may have a lower temperature in the nose due to extreme vasoconstriction). In the future, such phenomena must be focused on to ensure robust ROI segmentation.

However, our study had some limitations. Because of the fixed camera position at the foot of the bed, nostril regions were not tracked when patients moved to a lateral sleeping position, or when patients were agitated with strong urges to move. Additionally, with changing breathing patterns, such as extreme tachypnea or shallow breathing, the approach had difficulties distinguishing between inspiration and expiration.

In total, the IRT-based algorithm performed in a robust and reliable manner in a postoperative clinical setting, without disturbing postoperative care. In particular, the advantages of IRT, such as independence from a light source and the ability to handle motion artefacts, make it interesting for prospective daily use. Additionally, the reproducibility of IRT measurements is high when quality assurance (e.g., including data protocols) is given [34,35,36,37].

## 5. Conclusions

According to the current state of relevant studies as well as our experience, IRT may be a promising technique for contactless BR estimation. Our results showed that a feasible and reliable use of the presented approach was possible, even under challenging conditions in a postoperative clinical setting in the PACU. Furthermore, a close correlation between the IRT-based approach and the GT were obtained, especially for low and normal breathing frequencies. Further analysis should focus on the inferior performance of the algorithm for higher breathing rates. It is possible that a modification or development of the algorithm may improve the correlation between the IRT-based approach and the GT under this condition. Additionally, the recognition of shallow breathing or apnea must be examined in further studies. A fusion of newly-developed parameters (e.g., open-mouth respiration or shoulder and thorax movement) may improve the algorithm for further clinical studies.

## Figures and Tables

**Table 1:** Algorithm performance for different breathing rate (BR) categories.

Category	BR Range	Number of Datasets	Spearman’s Rho Correlation	*p* Value
A	<12 bpm	6	0.845	0.034
B	12–15 bpm	17	0.651	0.005
C	>15 bpm	24	0.458	0.024

**Table 2:** Algorithm performance for insufflation with nasal cannula and with no insufflation.

Category	Number of Datasets	Spearman’s Rho Correlation	*p* Value
No Insufflation	20	0.774	<0.001
Insufflation	27	0.690	<0.001

**Table 3:** Performance of the algorithm for each patient (n.a.; not applicable).

Patient	Time Point	BR (bpm)		Relative Error	Insufflation	Region of Interest (ROI)—Fraction of Image (%)	Breathing Pattern/Patient Movement
		Ground Truth (GT)	Infrared Thermography (IRT)				
1	1	16.00	11.54	0.28	no	0.03	shallow
	2	14.00	13.68	0.02	no	0.06	normal
2	1	24.00	n.a.	n.a.	no	n.a.	shallow
	2	16.00	16.03	0.00	no	0.07	normal
3	1	16.00	12.54	0.22	no	0.09	normal
	2	18.00	n.a.	n.a.	no	n.a.	movement
4	1	20.00	n.a.	n.a.	no	n.a.	movement
	2	12.00	n.a.	n.a.	no	n.a.	movement
5	1	14.00	13.48	0.04	no	0.20	normal
	2	14.00	12.35	0.12	no	0.20	normal
6	1	18.00	17.11	0.05	yes	0.13	normal
	2	20.00	18.64	0.07	yes	0.14	normal
7	1	16.00	12.60	0.21	no	0.16	shallow/movement
	2	16.00	n.a.	n.a.	no	n.a.	movement
8	1	20.00	15.94	0.20	yes	0.25	irregular
	2	20.00	16.49	0.18	yes	0.21	irregular
9	1	15.00	15.68	0.05	no	0.12	normal
	2	18.00	17.50	0.03	no	0.10	normal
10	1	15.00	14.72	0.02	yes	0.12	normal
	2	14.00	13.10	0.06	yes	0.13	normal
11	1	17.00	16.00	0.06	yes	0.08	normal
	2	11.00	11.17	0.02	yes	0.07	normal
12	1	12.00	11.01	0.08	no	0.18	normal
	2	12.00	10.49	0.13	no	0.14	normal
13	1	17.00	17.37	0.02	no	0.12	normal
	2	10.00	10.01	0.00	no	0.20	normal
14	1	18.00	15.45	0.14	no	0.10	normal
	2	16.00	15.85	0.01	no	0.19	normal
15	1	16.00	15.51	0.03	yes	0.14	normal
	2	16.00	15.04	0.06	yes	0.06	normal
16	1	20.00	n.a.	n.a.	yes	n.a.	no signal
	2	18.00	15.72	0.13	yes	0.08	normal
17	1	10.00	n.a.	n.a.	no	n.a.	movement
	2	12.00	13.80	0.15	no	0.24	normal
18	1	20.00	14.90	0.26	no	0.05	irregular
	2	18.00	16.88	0.06	no	0.07	normal
19	1	10.00	9.02	0.10	yes	0.06	normal
	2	12.00	11.48	0.04	yes	0.05	normal
20	1	16.00	12.68	0.21	yes	0.04	irregular
	2	12.00	12.09	0.01	yes	0.05	normal
21	1	16.00	11.35	0.29	yes	0.13	apnea
	2	16.00	10.08	0.37	yes	0.07	apnea
22	1	12.00	11.26	0.06	yes	0.06	normal
	2	16.00	15.05	0.06	yes	0.04	normal
23	1	20.00	7.93	0.60	yes	0.10	apnea
	2	10.00	9.43	0.06	yes	0.07	normal
24	1	10.00	10.38	0.04	yes	0.16	normal
	2	13.00	9.79	0.25	yes	0.13	apnea
25	1	n.a.	n.a.	n.a.	no	n.a.	lateral position
	2	n.a.	n.a.	n.a.	no	n.a.	lateral position
26	1	20.00	16.34	0.18	yes	0.09	movement
	2	12.00	11.31	0.06	yes	0.07	normal
27	1	12.00	9.33	0.22	no	0.06	movement
	2	8.00	7.63	0.05	no	0.11	normal
28	1	14.00	11.35	0.19	yes	0.08	normal
	2	12.00	10.83	0.10	yes	0.09	normal
